# The Effect of Family-centered Care on Management of Blood Glucose Levels in Adolescents with Diabetes

**Published:** 2015-07

**Authors:** Fatemeh Cheraghi, Farshid Shamsaei, Sayyedeh Zohreh Mortazavi, Abbas Moghimbeigi

**Affiliations:** 1 Chronic Diseases (Home Care) Research Center, Department of Nursing, School of Nursing and Midwifery, Hamadan University of Medical Sciences, Hamadan, Iran;; 2 Behavioral Disorders and Substance Abuse Research Center, Department of Nursing, School of Nursing and Midwifery, Hamadan University of Medical Sciences, Hamadan, Iran;; 3 Department of Nursing, School of Nursing and Midwifery, Hamadan University of Medical Sciences, Hamadan, Iran;; 4 Modeling of Noncommunicable Disease Research Center, Department of Biostatistics and Epidemiology, School of Health, Hamadan University of Medical Sciences, Hamadan, Iran

**Keywords:** Adolescent, Diabetes Mellitus Type 1, Family

## Abstract

**Background:**

Responsibility for diabetes management tasks must shift from caregivers to adolescents as adolescents grow older. Also, family-centered care is a way to provide efficient care for them at home. This study aimed to identify the effect of family-centered care on management of blood glucose levels in adolescents with type 1 diabetes mellitus (T1DM).

**Methods:**

This is a Pre-experimental study with a pre- and post-test design. The participants consisted of forty adolescents with T1DM, aged between 10-14 years, with their caregivers who were selected through simple random sampling from Hamadan Diabetes Research Center in Iran in 2013. The sample was divided into four similar groups. Educational sessions were conducted for each group for 30 to 40 minutes. Data collection tools were “Supervisory Behaviors of Caregiver” (SBC), “Management Behaviors of adolescents” (MBA) questionnaires, and the “Blood Glucose Levels Record Sheet”. Data were analyzed using SPSS 19 and based on descriptive statistics, Kolmogorov-Smirnov, paired t-test and Pearson coefficient.

**Results:**

There was a significant difference between the subjects’ MBA and SBC mean scores before (110.17±26.6) and after (134.6±1.28) intervention in four domains: “blood glucose testing”, “insulin therapy”, “meal plan” and “physical activity” (P<0.001). There were significant differences between the mean levels of recorded blood glucose during a week before and after intervention and between the mean levels of Glycated Hemoglobin level (HbA1c) before (8.4±1.12) and three months after (7.78±1.2) it (P<0.001). Pearson coefficient showed a positive relationship between the supervisory behaviors of caregivers with management behaviors of adolescents before and after the intervention (P<0.001).

**Conclusion:**

Empowering adolescents with T1DM and their caregivers in home-centered care could improve diabetic adolescents’ management of blood glucose levels and reduce their HbA1Clevels. Therefore, Family-centered care could provide for better regime adherence at home.

## Introduction

Diabetes mellitus is a serious disease with potentially devastating complications and is common among all age groups worldwide.^1^ After asthma, Diabetes is the second most common chronic disease during childhood.^2^ It disturbs children and families’ lifestyles, personality and mental, social, and economic conditions.^3^ The prevalence of type 1 diabetes mellitus (T1DM) is increasing in children all over the world.^4^ Approximately, one out of every 300 to 500 children under 18 years suffers from diabetes. Its prevalence in school-age children is 1.9 per thousand. In the United States, one out of every 400 children and adolescents has diabetes.^5^ However, in Iran, the annual incidence of T1DM has been estimated at 3.7 cases per one hundred thousand individuals.^6^ All over the world, this figure varies from 1 to 35 cases per one hundred thousand children under 14 year old.^7^

Like other chronic diseases, diabetes is a heavy burden for families and society. Medical costs for people with diabetes are two to three times higher than non-diabetics.^6^ Besides increasing treatment costs, the short- and long-term complications of T1DM cause serious problems in the life of children and their families. If diabetes is not controlled properly, vascular changes will occur in less than 3 years after diagnosis; however, adequate control can postpone these changes for up to 20 years and even more.^3^ Hypoglycemia, hyperglycemia and ketoacidosis are among the most common and important complications of diabetes in childhood and sometimes necessitate hospitalization. Other serious physical complications include visual, renal, cardio-vascular and neural disorders, which can result in blindness, severe kidney failure, heart or stroke attack and amputation.^8^ In order to avoid the complications of diabetes and reduce the risk of mortality related to T1DM, the patients require special and long-term care.^9^ Through adequate control of diabetes, the incidence rate of retinopathy, nephropathy and neuropathy were reduced as much as 76, 54 and 60 percent, respectively.^10^

The definition of management of diabetes is to keep blood glucose at appropriate levels.^11^ For that to happen, a T1DM patient is required to have daily injections of insulin, measure blood glucose, plan regular activity programs and comply with specific meal plans in order to appropriately stabilize blood glucose level and achieve optimal metabolic status. However, for children of all ages, it is a difficult and tedious process and the close and direct supervision of the family is required.^12^

Children, especially very young children, are unable to do many of the tasks related to the management of diabetes properly.^13^ In early adolescence, the beginning of the hormonal changes caused by puberty creates a natural resistance to insulin in the body. Sometimes, the onset of juvenile diabetes is between 10-14 years.^3^ In Addition, peer pressure, fear of difference, desire for independence, and separation from parents can hinder an accurate management of diabetes and cause blood glucose fluctuations.^14^ Studies have revealed that supervision and cooperation of parents in managing diabetes during childhood and adolescence result in better metabolic control.^15^^,^^16^ Furthermore, evidence from some studies show that family-based interventions have been effective with regard to blood glucose and metabolic control in children with T1DM.^17^ Therefore, in addition to caring, another duty of pediatric nurses is advocate the participation of family members to follow up the therapeutic regimen of children with T1DM at home.^3^ This important issue has been conceptualized in the form of family-centered care.

Family-centered care, as one of the main concepts of pediatric nursing, emphasizes support, increasing the knowledge of parents and children and the continuity of care for children with special needs.^18^ The main goal of family-centered care is preserving the integrity of families and providing unique care for diabetic children at home and in the society.^3^ Dimensions of family-centered care include caring for the child based on the family framework, facilitating the participation of parents in caring, identifying and reinforcing the strengths of the family; caring for the child based on his/her age, providing information for children and their families, acknowledging the uniqueness of each family and designing flexible and effective health care plans for each family.^4^ It should be highlighted that the involvement of families in caring for children and adolescents with T1DM is important. Prevention is an important component of the treatment paradigm, because tight glycemic control delays the onset and progression of complications of insulin-dependent diabetes mellitus (IDDM). Also, the health of children with diabetes can be promoted through family members’ participation in and supervision of their treatment at home. Therefore, they and their families will require continuing and lifelong education and training.^3^ The purpose of the current study was to identify the effect of family-centered collaborative care on management and appropriate control of blood glucose in adolescents with T1DM aged between 10 to 14 years. Since we intended to examine cross-sectional associations between responsibility sharing, diabetes management, and glycemic control in adolescents with type 1 diabetes and their caregivers, we hypothesized that more caregiver responsibility would correlate with more frequent diabetes management and lower A1c values.

## Materials and Methods

This Pre-experimental study had a pre- and post-test single-group design, and was conducted between February 2013 and June 2014. The sample was recruited from adolescents that had records in Hamadan Diabetes Research Center, Hamadan, Iran. The sample size was estimated based on the study of Rezai et al.,^19^ with a confidence level of 0.95 and test power of 80 percent. Thirty-seven adolescents were to be selected, but with regard to the risk of loss, 40 adolescents aged between 10 and 14 years together with their caregivers were selected through the simple random sampling method.

$$n=\frac{{\overset{-}{p}(1-\overset{-}{p})(z_{1-\frac{\alpha }{2}}+z_{1-\beta })}^{2}}{{\left(p_{1}-p_{2}\right)}^{2}}$$

$$\overset{-}{p}=\frac{p_{1}+p_{2}}{2}$$

*p*_1_=0.467

*p*_2_=0.767

$$1-\frac{\alpha }{2}=0.975$$

After assigning a number to each case that met the study inclusion criteria, the researchers selected the sample based on a table of random numbers.  Inclusion criteria included: being aged between 10-14 years, being at least six months post-diagnosis; not having a serious concurrent medical illness (e.g. presently hospitalized) or any other chronic diseases; nonparticipation of the adolescents or caregivers in other formal diabetic training programs within the month prior to the study; caregivers’ being literate, not having been diagnosed with major psychiatric illnesses, and  not having had any previous first-hand or second-hand experience of diabetes..

Research instruments had to be developed to examine how youths with type 1 diabetes and their caregivers share responsibilities related to diabetes management. Because diabetes self-care is multidimensional, especially for adolescents, it is necessary that each component be assessed. Diabetes family-centered care includes a range of activities and adults’ supervision (e.g., monitoring of blood glucose, insulin therapy, eating a low-saturated-fat diet, and children’s physical activity). Therefore, well-established instruments were needed to study these different components. Based on the literature review, research instruments were developed by the researchers: “Demographic Data”, “Supervisory Behaviors of Caregiver” (SBC), “Management Behaviors of adolescents (MBA) with T1DM” questionnaires and the “Daily log of blood sugar levels for diabetics” as recommended by American Diabetes Association.^20^

The SBC questionnaire was developed based on the work of Harris et al. (2000) and Schafer et al. (1986).^21^^,^^22^ The SBC questionnaire was completed by the caregivers and had 32 items on a five-point Likert scale (from never to always) and addressed four areas of monitoring and supervision, namely blood glucose testing, insulin therapy, meal plan, and the child’s physical activity.

The MBA questionnaire was developed based on the studies of Harris et al. (2000) and La Greca (1995).^21^^,^^23^ The self-report MBA questionnaire, completed by adolescents, also had 32 items on a five-point Likert scale (from never to always) and addressed the four areas of blood glucose testing, insulin therapy, meal plan and doing physical activity. For both questionaires, the score range was between 32 -160. At the end of the demographic section, two items were added to note the last measured Glycated haemoglobin level (HbAlc) before the intervention and three months after it with the date of the blood test.

In the current study, the items of both questionnaires were translated into Farsi. Then again, the Farsi versions were translated into English by a professional translator. After that, the new English versions were compared with the original ones. The face and content validities of the instruments were confirmed by 10 nursing faculty members at Hamadan University of Medical Sciences, two pediatric endocrinologists and 6 children with diabetes and their parents that were not included in the sample. For the SBC and MBA questionnaires, content validity ratio (*CVR*) and content validity index (*CVI*) were calculated. In both cases, CVR was higher than 83 percent and CVI was higher than 80 percent, which were the best percentages. Pearson’s correlation coefficients (PCC) were used to evaluate the parent–child agreement; p-values less than .05 were interpreted as significant. Good correlation (r=0.708 before and r=0.748 after intervention for the overall sample) was found to exist between parents and adolescents.

Test-retest reliability in the pilot study with 15 children with diabetes and their parents, who were not included in the sample, with a two-week interval was found to be 0.87. The internal consistency reliability of the questionnaires was calculated with Cronbach’s alpha coefficient. Cronbach’s alpha coefficient of the overall SBC questionnaire was α=0.91; it was α=0.68 for supervision on blood glucose testing, α=0.74 for supervision on therapy insulin, α=86 for supervision on meal plan and α=0.75 for supervision on physical activity subscales. Cronbach’s alpha for the overall MBA questionnaire was α=0.91; it was α=0.74 for blood glucose testing, α=0.93 for insulin therapy, α=0.91 for meal plan and α=0.84 for physical activity subscales.

*Procedure*

The second researcher explained and clarified the study’s purpose and anonymity policies to each participant (adolescents and caregivers). Participants had the option to withdraw at any time. Then, written consent was taken from the participants. After checking with the Diabetes Research Center and the subjects, the researchers chose the time and location of training classes and the participants were invited to the educational sessions. None of the participants refused. Since coordination, education and answering questions in smaller groups are easier, four groups with 20 participants (10 adolescents with T1DM aged 10-14 years along with their caregivers) were formed. Four educational and practical training sessions were held for each group (total sessions were 16). Each session lasted between 30-40 minutes. The classes included lectures, group discussions, demonstration and role playing based on the content.  At the end of the courses, each participant was given the educational booklet. A simplified version of the same educational content was used for the classes. The content of the sessions and booklet included basic information about diabetes (e.g. physiology, symptoms, complications, caring, follow-up), empowering for self- and family-centered care concerning blood glucose monitoring, using a glucometer, insulin therapy, meal plans, physical activity and exercise. 

One week before and one month after the educational sessions, the subjects completed the scales. Also, the caregivers were asked to write down the adolescents’ blood glucose levels in “Blood Glucose Levels Record Sheet” before and after breakfast, lunch and dinner, and before bedtime for a week before and after intervention. Each adolescent’s last Glycated Hemoglobin level (HbA1c) before intervention was recorded. Three months after intervention, it was checked again by telephone. The patients’ blood samples were obtained by fingerstick at their regular clinic appointments and the same central laboratory. 

*Ethical Consideration*

All procedures were approved by Hamadan University of Medical Sciences Ethics Committee (N0. 911215481-P/16/35/4697). Written informed consents were obtained from the subjects.

*Data Analysis*

Statistical analysis was carried out using SPSS version 19 (SPSS, Inc., Chicago, IL, USA). Analysis was based on descriptive statistics and paired t-test, Kolmogorov-Smirnov and Pearson correlation coefficient at 95% confidence level. 

## Results

The majority of the adolescents was female (57.5%), first born (35%), studying in primary school (52.5%), and had signs of puberty (57.5%). The adolescents’ ages ranged from 10-14 years, with the mean of 11.8 and SD of 1.44 years. Weights ranged from 24-58 kilograms, with the mean of 40.16 and SD of 7.81 kg. More than half of the surveyed adolescents (52.5%) had T1DM for more than 37 months. The mean of hospitalization due to diabetes during the previous year was 1.85 times (SD=1.02) (the frequency range was from 0-5 times). The means of the ages of the fathers and mothers were 44.4 (SD=6.34, ranging between 33-57 years) and 39.8 (SD=5.90, ranging between 30-57 years), respectively. The majority of the fathers (25%) had high school diploma and were self-employed (45%). The majority of the mothers (30%) had high school diploma and were homemakers (95%). The majority of the surveyed caregivers (80%) were mothers (Table 1). The mean of HbA1c levels was 8.40 (SD=1.12) before, and 7.78 (SD=1.16) percent three months post-intervention. The mean of one-week blood glucose level was 185.27 (SD=47.25) before, and 164.07 (SD=34.72) after intervention.

**Table 1:** Sociodemographic characteristics of diabetic adolescents

**Variable**	**Mean** **±** **SD**	**Range**	**n (%)**
Age (years)	11.81±1.51	10-14	
Weight (kg)	40.20±7.81	24-58	
Hospitalization (months)	1.80±1.02	1-5	
Age of fathers (years)	44.41±6.32	33-57	
Age of mothers (years)	39.81±5.91	30-57	
Gender			
Male			17 (42.5)
Female			23 (57.5)
Signs of puberty			
Yes			23 (57.5)
No			17 (42.5)
Duration of diabetes (months)			
12<			7 (17.5)
12-24			7 (17.5)
25-36			5 (12.5)
37>			21 (52.5)
Rank of birth			
First			14 (35)
Second			10 (25)
Third			8 (20)
Fourth and next			8 (20)
Education of children			
Dropout			1 (2.5)
Primary school			21 (52.5)
Junior high school			14 (35)
High school			4 (10)
Main caregiver			
Father			32 (80)
Mother			5 (12.5)
Other family members			3 (7.5)
Marital status (parent)			
Married			35 (87.5)
Single			2 (5)
Death of a parent			3 (7.5)
Work status (father)			
Government employee			6 (15)
Worker			11 (27.5)
Self-employed			18 (45)
Unemployed			2 (5)
Retired			3 (7.5)
Work status (mother)			
Government employee			2 (5)
Homemaker			38 (95)

Kolmogorov-Smirnov test demonstrated that there was a normal distribution of the mean scores of subjects in all the subscales (P<0.05). Paired t-test revealed a significant difference between the mean scores of the adolescents’ management behaviors in the domains of insulin therapy, blood glucose testing, meal plan and doing physical activity (P<0.001) (Table 2). It also showed a significant difference between the mean scores of the caregivers’ supervision on adolescents’ blood glucose testing, insulin therapy, meal plan and physical activity before and after intervention (P<0.001) (Table 3). There was a significant difference between the means of the recorded blood glucose levels during the week before and the week after intervention, as well as between the means of HbA1c levels before and three months after intervention (P<0.001) (Table 4).

**Table 2:** Management behaviors of adolescents with T1DM pre- and post-intervention

**Management Behaviors**	**Pre-int.**	**Post-int.**	**t**	**P**
	**Mean±SD**	**Mean±SD**		
Blood glucose testing	14.89±4.15	18.40±2.15	7.44	<0.0001
Insulin therapy	36.78±10.12	37±10.26	7.43	<0.0001
Meal plans	41.20±11	53.3±6.3	9.72	<0.001
Physical activity	15.51±3.15	25.8±3.3	12.18	<0.0001
Total	110.17±26.6	134.6±1.28	10.65	<0.001

**Table 3:** Supervisory Behaviors of Caregivers pre- and post-intervention

**Supervisory Behaviors**	**Pre-int.**	**Post-int.**	**t**	**P**
	**Mean±SD**	**Mean±SD**		
Supervision on blood glucose testing	15.51±3.15	18.4±2.03	7.41	<0.0001
supervision on insulin therapy	36.30±7.6	46.15±3.45	10.86	<0.001
supervision on meal plans	46.02±9.8	54.9±5.43	8.90	<0.001
supervision on physical activity	20.57±5.14	27±2.60	10.19	<0.0001
Total	119.4±21	145.5±11.4	13.83	<0.0001

**Table 4:** Glucose control pre- and post-intervention

**Variable**	**Pre-int.**	**Post-int.**	**t**	**P**
	**Mean±SD**	**Mean±SD**		
BG in a week	185.3±47.25	164±34.7	3.97	<0.0001
HbA1c level	8.4±1.12	7.78±1.2	5.23	<0.001

**Table 5:** Correlation between main variables pre- and post-intervention

**Variable**	**Pre-int.**		**Post-int.**	
	**r**	**P**	**r**	**P**
Blood glucose testing and the supervision	0.390	<0.013	0.550	<0.0001
Insulin therapy and the supervision	0.478	<0.002	0.579	<0.0001
Meal plan and the supervision	0.704	<0.0001	0.748	<0.0001
Physical activity and the supervision	0.658	<0.0001	0.627	<0.0001
Management behaviors of adolescents and supervision of caregivers (Total)	0.708	<0.0001	0.748	<0.0001

The adolescents’ management behaviors significantly correlated with their caregivers’ supervisory behaviors (P>0.001); also, there was a positive correlation in the domains of insulin therapy before (P>0.01) and after (P>0.001) intervention, blood glucose testing (p<0.01), meal plan, and doing physical activity (P>0.001) before and after intervention (Table 5).

## Discussion

The present study confirmed the assumption that family-centered intervention, by empowering both 10-14-year-old adolescents with T1DM and their caregivers, could improve the management of blood glucose levels in childhood at home. In other words, training, with an emphasis on sharing responsibilities between adolescents with T1DM and their families, was a useful way to provide a healthy life and better regime adherence. This finding was consistent with the results of other studies that showed family-centered care promoted the level of health in children,^17^^,^^24^ and facilitated blood glucose control and diabetes management in adolescents with T1DM.^17^ In their study, Pereira et al. (2008) confirmed the relationship between family support and children’s commitment to diabetic treatments.^25^ Faulkner and Chunk (2007) showed that nurses should promote the abilities of adolescents with T1DM and their families and improve their interaction for better caring and follow-up treatment at home.^3^

The management precision of the caregivers and adolescents with T1DM improved in testing blood glucose. In clinical trial study, Rosal et al. (2005) found that, after the training intervention, the case group monitored their blood glucose level more efficiently than the control one.^26^ Also, more supervision from parents in the management of diabetes increases the number of times teenagers with diabetes measure their blood glucose levels.^13^

The training intervention improved the adolescents’ insulin administration and insulin-dose adjustment and the caregiver’s monitoring and supervision of the performance of adolescents with T1DM. These results were consistent with the study of Silverstein et al. (2005): their study showed that teaching self-care to patients with T1DM was associated with reduced complications from insulin injection, such as atrophy of the injected area. Parental monitoring and supervision on insulin therapy led to treatment adherence in adolescents with T1DM.^1^

After the educational intervention, the children’s dietary behaviors and the caregivers’ supervision on their diet improved. Family-centered care could create a supportive family environment for empowering individuals and families in improving a patient’s meal plan.^27^ Based on the study of Fisher et al. (2010), there was a relationship between family supervision and improvement in daily diet meal and exercise planning among Spanish adolescents with T1DM.^28^ Moreover, a meal plan with three meals and three snacks prevents hyperglycemia and hypoglycemia in patients with T1DM.^1^ However, in another study, after the training intervention, the participants still misunderstood their disease and how to manage diabetics’ diet.^29^

As with the study of Yates et al. (2009),^30^ after the educational intervention, the knowledge of the caregivers and adolescents with T1DM about the importance and positive impact of physical activities on blood glucose control increased. In the study of Rosal et al. (2005), the educational intervention improved metabolic control of women with T1DM and the results revealed an increase in daily physical activities of the case group compared to the control one.^26^

The results showed a decrease in the mean level of recorded blood glucose during the week after intervention. Therefore, family-centered empowerment of the adolescents and their caregivers could reduce the blood glucose levels in adolescents with T1DM. These results are consistent with the results of the study of Mohammad et al. (2012) that indicated a significant relationship between daily and weekly measuring and blood glucose control.^31^ Therefore, parents’ monitoring increased the children’s commitment to measuring their blood glucose and led to better control of blood glucose.

In the present study, three months after intervention, the HbA1c level decreased. The study of McBroom and Enriquez (2009) showed family-centered care reduced HbA1c level and increased family dynamics. They concluded that family-centered care improved the metabolic status of children with T1DM.^17^ Teaching dietary self-management could decrease the mean level of HbA1c in children from 8.50 (SD=0.77) to 7.92 (SD=0.74) percent. Besides, parental supervision reduced HbA1c level and led to better metabolic control.^28^^,^^29^

It is accepted that children aged 9 years old and above have direct responsibility for their treatment and self-care and parents must begin to transfer responsibilities for diabetes management to children in later childhood; also, there was a statistically positive relationship between the management behaviors of the caregivers and adolescents with T1DM. In other words, promoting the caregivers’ ability to supervise and monitor the performance of adolescents with T1DM could promote adolescents’ management behaviors and enhance their accuracy in regimen adherence, including blood glucose testing, insulin therapy, meal plans and physical activities.  Our findings show that adolescents aged 10 to 14 years still required the supervision of the family for effective management of blood glucose levels. Consequently, family-centered care led to better regime adherence and control of blood glucose in adolescents with T1DM.^1^

In sum, the results of this study suggest that, although adolescents with type 1 diabetes can gain greater autonomy in their self-care, they still need caregivers’ supervision in the management of their diabetes. Based on family-centered care, it is necessary that the responsibility sharing of the caregiver be explicit and clearly identified by the adolescent. Explicit responsibility sharing appears to be particularly important for direct management tasks. Thus, clinic-based and nursing research interventions for adolescents must include caregivers’ and adolescents’ responsibility sharing in order to promote diabetes management and glycemic control for adolescents with type 1 diabetes. 

This study has limitations. First, we relied on self-report measures for responsibility sharing in management of diabetes. However, we found that adolescent self-management and caregiver responsibility were important correlates of diabetes management, but it is possible that this finding differs from what actually goes on in the daily management of type 1 diabetes. Second, we didn’t use a control group because there was a limitation for sample selection: the number of adolescents and their parents in Hamadan Diabetes Center that met the inclusion criteria and were willing to cooperate in the research was limited. Therefore, the findings should be generalized with caution. Family-centered care studies with control groups should be planned for a closer examination of responsibility sharing of caregivers and adolescents with type 1 diabetes with regard to management of treatment regimens and predicting health status. 

## Conclusion

Family-centered intervention with an emphasis on the participation and empowering of family members can improve the supervision of caregivers over adolescents with diabetes and their blood glucose. Additionally, it can empower adolescents with T1DM in regimen adherence, including blood glucose testing, insulin therapy, meal plan and physical activity.  Also, it reduces the levels of blood glucose and HbA1c. As a result, family-centered care improves the management of blood glucose levels in adolescents with T1DM. . 
